# Complete Genome Sequence of *Vitreoscilla filiformis* (ATCC 15551), Used as a Cosmetic Ingredient

**DOI:** 10.1128/genomeA.00913-17

**Published:** 2017-08-24

**Authors:** Sandy Contreras, Pierre Sagory-Zalkind, Hélène Blanquart, Agnès Iltis, Stanislas Morand

**Affiliations:** a Genoscreen, Lille, France; b Genostar, Montbonnot Saint Martin, France; c L’Oréal, Research and Innovation, Aulnay-sous-Bois, France

## Abstract

We report the first complete genome sequence of a *Vitreoscilla filiformis* strain (ATCC 15551) that is used in the cosmetic industry as *Vitreoscilla* ferment. The assembled genome consisted of one chromosome and two plasmids. These data will provide valuable information and important insights into the physiology of this filamentous organism.

## GENOME ANNOUNCEMENT

The nonpathogenic aerobic Gram-negative *Vitreoscilla filiformis* bacterium was named on the basis of its colorless gliding filamentous morphology (1). In the Pyrénées mountains, *V. filiformis* was spotted in spa muds used for skin cures and subsequently fermented at an industrial scale for more than 20 years to generate a bacterial lysate introduced into emollients. Studies have shown that this *V. filiformis* extract improved atopic dermatitis (2–5) by increasing keratinocyte antioxidant manganese superoxide dismutase (MnSOD) mitochondrial content (6) through a still-unknown mechanism. Currently, only two *Vitreoscilla* scaffold genomes are available (*Vitreoscilla stercoraria* and *Vitreoscilla massiliensis*). Here, we present the first complete genome sequence of a *V. filiformis* strain (ATCC 15551).

Genomic DNA was isolated using the Gentra Puregene kit (Qiagen) following the manufacturer’s guidelines. Sequencing was performed by Genoscreen on a PacBio RS II system (Pacific Biosciences), following construction of a 10-kb SMRTbell library, and on a HiSeq 2500 system (Illumina), following construction of a 2-kb Nextera XT library. The generated long-read sequences (1,842,560,472 nucleotides; 116,681 reads) were *de novo* assembled using the single-molecule real-time (SMRT) analysis software version 2.3.0 followed by Circlator version 1.4.1 for circularization (7). The assembly quality was checked using 250-base paired-end short-read sequences (595,503,983 nucleotides; 1,250,485 reads) with BOWTIE2 version 2.1.0. The assembled genome comprised 3,765,551 bp, consisting of one chromosome (3,484,895 bp) and two plasmids, pVF1 (240,640 bp) and pVF2 (40,016 bp). Structural and functional annotations carried out by Genostar using a proprietary pipeline identified 6 rRNAs (5S, 16S, and 23S), 61 tRNAs, and 3,588 protein-coding sequences, of which 2,409 (67%) were annotated with known biological functions and 1,179 (33%) encode hypothetical proteins or uncharacterized proteins.

The overall genome GC content (63.5%) fits that of a previous determination, ranging from 59 to 63% (1). The *V. filiformis* genome size (3.77 Mb) differs from those of the *Vitreoscilla stercoraria* (5.16 Mb, 43.9% GC) and *Vitreoscilla massiliensis* (7.43 Mb, 49.4% GC) genomes sequenced to date. The *Vitreoscilla* hemoglobin gene used in biotechnology processes is not present in the *V. filiformis* genome (8, 9). The *V. filiformis* bacterium is known to produce poly-β-hydroxybutyrate (PHB) granules that can accumulate to greater than half of its dry weight (1). Genome analysis confirmed the presence of the PHB operon (*phbA*, *phbB*, and *phbC*) as well as its regulator *phaR*. Interestingly, the PHAST algorithm (10) revealed that the chromosome comprises 5 similar prophage loci of ca. 38 kb with no resemblance to known phages. Moreover, a 10.5-kb region that contains transporter-, permease-, and transposase-encoding genes is duplicated twice on the chromosome and once on pVF2. Two large >7-kb-long clustered regularly interspaced short palindromic repeat (CRISPR) arrays composed of 117 and 129 repeats occur on the chromosome and pVF1, respectively.

Strohl et al. (1) reported that, unlike *V. stercoraria*, *V. filiformis* uses glucose, citrate, lactate, aspartate, glutamate, succinate, or acetate as sole carbon and energy sources and nitrate (NO_3_^−^) as the sole nitrogen source and is resistant to neomycin. The mining of the complete genome sequence of *V. filiformis* will provide insights into the physiology of the *Vitreoscilla* genus, facilitating control of its industrial fermentation.

### Accession number(s).

The complete genome sequence is available in GenBank under the accession numbers CP022423 to CP022425. The version described in this paper is the first version.
